# Shorter telomere length in peripheral blood leukocytes is associated with childhood autism

**DOI:** 10.1038/srep07073

**Published:** 2014-11-17

**Authors:** Zongchang Li, Jinsong Tang, Hong Li, Shan Chen, Ying He, Yanhui Liao, Zhen Wei, Guobin Wan, Xi Xiang, Kun Xia, Xiaogang Chen

**Affiliations:** 1 Institute of Mental Health, the Second Xiangya Hospital of Central South University, Changsha, Hunan, China; 2 Institute of Genomic Medicine, Wenzhou Medical University, Wenzhou, Zhejiang, China; 3 Department of Women's Health Care, The Affiliated Shenzhen Maternal and Child Health Care Hospital, Nanfang University of Medical Science, Shenzhen, Guangdong, China; 4 BGI Ark Biotechnology Co., Ltd., Shenzhen, Guangdong, China; 5 The State Key Laboratory of Medical Genetics, Central South University, Changsha, Hunan, China; 6 Key Laboratory of Psychiatry and Mental Health of Hunan Province, Central South University, Changsha, Hunan, China; 7 National Technology of Institute of Psychiatry, Central South University, Changsha, Hunan, China

## Abstract

Telomeres are protective chromosomal structures that play a key role in preserving genomic stability. Epidemiologic studies have shown that the abnormal telomere length in leukocytes is associated with some mental disorders and age-related diseases. However, the association between leukocyte telomere length and autism has not been investigated. Here we investigated the possible association between relative telomere length (RTL) in peripheral blood leukocytes and childhood autism by using an established real-time polymerase chain reaction method. We observed significantly shorter RTL in patients with childhood autism than in controls (p = 0.006). Individuals with shorter RTL had a significantly increased presence of childhood autism compared with those who had long RTL. In patients, we found that family training interventions have a significant effect on telomere length (P = 0.012), but no correlations between RTL and clinical features (paternal age, maternal age, age of onset, illness of duration, CARS score and ABC score) were observed in this study. These results provided the first evidence that shorter leukocytes telomere length is significantly associated with childhood autism. The molecular mechanism underlying telomere length may be implicated in the development of autism.

Human telomeres are nucleoprotein complexes that are composed of repetitive nucleotide sequences coated by shelterin proteins located at the end of linear chromosomes. The function of telomeres is to protect the chromosomes from degradation and fusion and maintain the genomic integrity^1^. The telomere length is maintained by the expression of telomerase in some human cells, such as stem cells and germ cells. However, in most normal somatic cells, the activity of telomerase is down-regulated and the telomeres progressively shorten with each cell division until the length reached is critically short, ultimately prompting cellular senescence and apoptosis^2^. Apart from cell division, telomere length is also influenced by many genetic and environmental factors. The rate of telomere attrition has been reported to be exacerbated by genetic and epigenetic variants^3^,^4^,^5^,^6^, oxidative stress^7^, smoking^8^ and socioeconomic status^9^. Multiple epidemiologic studies have indicated that accelerated telomere attrition in peripheral blood leukocytes (PBLs) is associated with the susceptibility of cancer^10^,^11^,^12^, cardiovascular disease^13^,^14^, shorter lifespan and a variety of age-related diseases^15^,^16^. Recently, increasing evidence has demonstrated that leukocytes telomere length is also associated with increased risk of some psychiatric diseases including schizophrenia, mood disorders and anxiety disorders^17^,^18^,^19^,^20^,^21^,^22^. However, the association between leukocytes telomere length and the risk of childhood autism has not been investigated.

Autism is a neurodevelopmental disorder characterized by social deficits, impaired communication and restricted or stereotyped behaviors and typically onset during childhood. Although, the pathology of childhood autism remains unclear, there has been strong evidence suggesting that inherited factors, nutritional factors, oxidative stress and inflammation contribute to this disorder^23^,^24^,^25^,^26^. Early twin studies indicated that autism is one the most heritable developmental disorders with a heritability estimate of about 90%^27^,^28^, and previous etiological research in autism has focused predominantly on genetic factors^29^. However, two more recent and large twin studies found a smaller genetic effect and a larger environmental effect on autism liability than earlier research^23^,^29^, which suggested a substantial role for shared environmental influences. Previous studies have demonstrated that telomeres dysfunction can increase genetic variation and cause genomic instability^30^,^31^, and can serve as an indicator to partially reflect both genetic predispositions and environmental burden. Considering that telomere shortening has been associated with some psychiatric diseases^17^,^18^,^19^,^20^,^21^,^22^, there is a possibility that dysfunctional telomeres might be involved in autism pathogenesis and that altered telomere length may be associated with childhood autism.

In the present investigation, we used a case-control study design to evaluate the association between leukocyte telomere length and childhood autism as well as with several clinical parameters of the disease.

## Results

### Subject characteristics

A total of 110 autism patients (male 98 and female 12) and 129 healthy controls (male 98 and female 31) were recruited in this study. The characteristics of all the subjects are presented in Table 1. There were age-matched between patient and control groups (p = 0.393), but there were significant differences in gender distribution (p = 0.008). The mean ages of patients with autism and controls were 56.98 ± 24.94 months and 59.82 ± 26.06 months, respectively. Consistent with previous reports, the patients with childhood autism included a significantly higher percentage of male than female (89.1% vs 10.9%).

### Association between RTL and childhood autism

**Figure 1:**
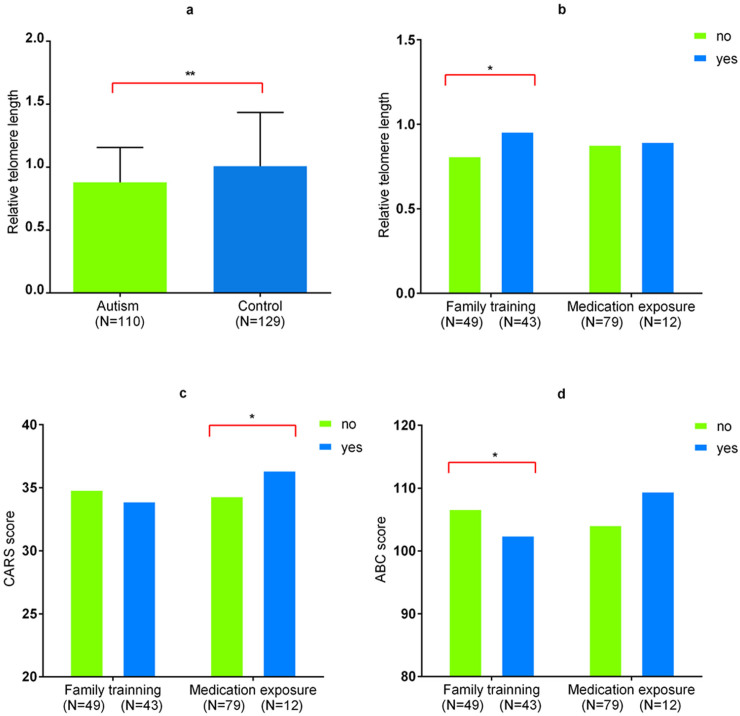
The relative telomere length,CARS score and ABC score in subjects among different groups. (A) Relative telomere length in patients with autism and healthy controls (mean ± SD). (B) Relative telomere length in patients with and without family training, and in patients with and without medication exposure. (C) CARS score in patients with and without family training, and in patients with and without medication exposure. (D) ABC score in patients with and without family training, and in patients with and without medication exposure. * P<0.05; **P<0.01.

Patients with autism had significantly shorter RTL than controls (0.88 ± 0.28 vs. 1.01 ± 0.43, p = 0.006, Figure 1a). No significant difference in RTL was observed between male and female subjects (P>0.05). In stratified analysis according to gender, the mean RTL from patients were significantly shorter than that from controls (0.86 ± 0.25 vs. 0.99 ± 0.44, p<0.013) in male subjects. However, in female subjects, there were no significant difference in RTL between patients and controls (1.01 ± 0.44 vs. 1.06 ± 0.38, p = 0.734), which was probably because of the small number of female patients (n = 12) in our study and the limited power to examine such associations.

We then performed an unconditional logistic regression analysis adjusting for age and gender to evaluate the association between RTL and the risk of autism (Table 2). When participants were divided into long and short groups according to the median RTL value of healthy controls, we observed a significantly increased presence of autism for individuals with shorter RTL (adjusted OR = 2.15; 95% CL, 1.25–3.71; p = 0.006; Hosmer-Lemeshow p = 0.60) compared with those with longer RTL. In addition, when participants were categorized further into three groups based on the RTL distribution of healthy controls, we found a significant dose-response relation between shorter RTL and increased presence of autism (p_trend_ = 0.006). That is, when using the first (longest) tertile as the reference group, the adjusted ORs for second and third tertile were 1.66 (95% CL, 0.82–3.36) and 2.59 (95% CL, 1.31–5.14), respectively (Hosmer-Lemeshow p = 0.58).

### Association of RTL with age and clinical characteristics

Pearson correlation analysis revealed a significant inverse correlation between RTL and age in controls (r = −0.30, p = 0.001). Similarly, there was a significant correlation between RTL and age in combined samples (cases and controls; r = 0.24, p<0.001). However, we failed to find the significant correlation between RTL and age in autism cases (r = 0.178, p = 0.064). In addition, the associations between RTL and clinical features in childhood autism are shown in Table 3. No significant associations were observed between RTL and clinical features (paternal age, maternal age, age of onset, illness of duration, CARS score and ABC score). However, we found an intriguing result: that among the subjects with childhood autism those who received family training interventions have significantly longer RTL than those without family training interventions (P = 0.012, Figure 1b). Patients with family training interventions also tended to have lower scores of clinical symptoms (Figure 1c, 1d). However, patients who had experienced medication exposure tended to have more severe symptoms than those without medication exposure (Figure 1c, 1d), and medication exposure has no effect on RTL (Figure 1b).

## Discussion

In the current study, we measured the leukocyte telomere length with quantitative PCR technique to investigate the different telomere status in patients with childhood autism and healthy controls. Our findings indicated that there was a significantly shorter leukocyte telomere length in patients with childhood autism than in healthy controls. Individuals with short leukocyte telomere length had a significantly higher presence of childhood autism than those with long leukocyte telomere length after adjusting for age and gender. Furthermore, we found that family training interventions had a significant effect on leukocyte telomere length. However, no correlations between RTL and clinical features (paternal age, maternal age, age of onset, illness of duration, CARS score and ABC score) in childhood autism were observed. To the best of our knowledge, this is the first study to explore the association between leukocyte telomere length and childhood autism.

The present study provides the insight that short telomere length in peripheral leukocytes is associated with childhood autism. There are several different possibilities for the relationships between short RTL and autism. The first possibility is that short telomeres might be a causal factor for autism progression. Previous studies of telomere biology demonstrated that telomeres are implicated in human polygenic diseases^10^,^17^,^18^, and that critically shortened telomere length can cause DNA damage and genomic instability^15^,^16^, which may be supposed to account for part of the increasing risk for developing autism. The second possibility is that short telomeres might be a result of the autism progression. The telomere length has been supposed to be negatively correlated with both inflammation activity and oxidative stress. Previous studies have suggested that inflammatory activity might accelerate the telomere shortening by increasing cell turnover^32^ and that telomeric DNA was highly sensitive to be damaged by the oxidative stress because of its high content of guanines^7^. Individuals with autism have elevated inflammatory activity and abnormal oxidative stress^33^,^34^, which make them susceptible to short telomeres. Finally, the third possibility is that the association between telomere length and autism might not be causal or reverse causal. They both could have originated by the same causal principle and be unrelated. For example, a specific stress could originate short telomeres and autism through independent pathways. This situation could explain the lack of correlation between telomere length and clinical parameters (CARS or ABC scores) in autism.

Consistent with previous studies^35^,^36^,^37^, this study demonstrated that leukocyte telomere length and age has a strong negative correlation in healthy controls and further supported to the concept of telomeres as a manifestation of biological aging. However, the association between leukocyte telomere length and age was not observed in the autism group. It is possible that the age effects are not detectable in the autism group because they already have shorter telomeres. Therefore, any additional age effect is not observed. Mixed findings have been drawn from many previous studies that investigated the relationship between telomere length and gender^38^,^39^,^40^,^41^. Furthermore, a recently published meta-analysis study has shown that these associations varied by measurement methods^38^. Southern blot analyses showed a significant difference but no differences were detected with either real-time PCR or Flow-FISH analyses. In our study, leukocyte telomere length was evaluated with real-time PCR and similar RTLs were found in males and females. Apart from measurement methods, a plausible explanation for the inconsistent results is that age, cell types, populations and other potential confounders including body mass index and epigenetic status might have affected the strength of the associations^38^,^42^,^43^. Therefore, more studies with adjustment for these confounders are needed to elucidate whether there are gender differences in telomere length.

It is interesting that family training interventions have an effect on RTL and are associated with patient's symptoms. There are several possible explanations for these intriguing findings. First, numerous studies have demonstrated that exposure to stress can accelerate telomere erosion^44^,^45^,^46^. Patients with family training interventions might experience less life stress and have a significant reduction of damage to telomeres. Second, family training interventions may be an effective therapy for autism and relatively protect telomeres from accumulating cellular damage associated with the illness. However, we found that medication exposure has no effect on RTL in autism. This finding was inconsistent with previous studies in other psychiatric patients showing medication treatment associated with changes in telomere length^47^,^48^. This conflict was probably due to the small number of medicated patients in our study and the limited power to examine such associations.

The major strength of our study is the unique and highly homogenous patient subjects. Autism spectrum disorders is a heterogeneous group of common developmental disorders including autism, Asperger's syndrome and pervasive developmental disorder—not otherwise specified (PDD-NOS). Our study is just restricted to childhood autism, excluding the patients with atypical autism and adult autism. Thus, any potential confounding effects yielded by different sub-diseases aetiology were eliminated, which may be efficient in this association study to evaluate the dynamic telomeres status in childhood autism. There are a few limitations to this study. First, the sample size was relatively small because of the rarity of patients with childhood autism. Then we were underpowered to conduct a further subgroup analysis. Second, we only measured telomere length in leukocytes from peripheral blood but not in the cells from brain tissue. However, leukocyte telomere length was significantly correlated with the telomere length in other somatic tissues^49^,^50^,^51^,^52^, and may serve as a surrogate for the telomeres length in other tissues. Furthermore, peripheral blood leukocytes are easily accessible. Thus, the measurement of telomere length in peripheral blood leukocytes is more practical in clinical research.

In conclusion, this is the first study to investigate the association between leukocytes telomere length and patients with childhood autism. Our results demonstrated a strong association between shorter leukocyte telomere length and childhood autism implying a possible molecular mechanism of telomeres implicated in autism pathology. Meanwhile, our results suggested that family training interventions may improve symptoms in autistic children and have potential impact on telomere erosion. Further studies with larger samples and prospective design are needed to confirm our finding, and functional analyses are also needed to reveal the underlying molecular mechanisms of telomeres in the etiology of autism.

## Methods

### Participants

Subjects with childhood autism were recruited from the Institute of Mental Health, Second Xiangya Hospital of Central South University and department of Children psychology, MCH Hospital of Shenzhen. One hundred and ten patients with childhood autism (ages from 3 to 14 years) were included in the study. Diagnosis was based on clinical evaluation by two senior clinicians and all cases met the international Diagnostic and Statistical Manual of Mental Disorders, fourth edition (DSM-IV) criteria for autism. To confirm and further evaluate the diagnosis, all patients were also assessed by using the childhood autism rating scale (CARS) and autism behavior checklist (ABC). Patients with childhood schizophrenia, Asperger's syndrome, Rett syndrome, Heller syndrome, pervasive developmental disorder not otherwise specified and fragile-X syndrome were excluded.

A total of one hundred and twenty-nine unrelated healthy controls (ages from 3 to 14 years) were recruited from community volunteers and were well age and geographical matched to the cases, avoiding problems of bias resulting from an inappropriate control population. The current mental status and history of mental disorders of the control subjects were evaluated by a senior psychiatrist. Control subjects were included in this study if they denied any history of mental disorders, neurological disorders, substance abuse and serious physical diseases. We did not check whether they had a family history of mental disorders.

The complete details of the entire study design and procedures involved were in accordance with the Declaration of Helsinki. All participants and their parents gave their written informed consent to participate in the study after the risks and benefits we discussed in detail. If the participants failed to fill out the consent form more than twice, then only their parents were asked to fill out the consent form on the patients' behalf. This study was approved by the ethics committee of the Second Xiangya Hospital of Central South University.

### Clinical assessment

The severity of autism was assessed by CARS, which rates the child on a scale from one to four in each of 15-items behavioral rating scale. The items are: relating to people; emotional response; imitation; body use; object use; listening response; fear or nervousness; verbal communication; non-verbal communication; activity level; level and consistency of intellectual response; adaptation to change; visual response; taste, smell and touch response and general impressions. Children with a total score above 30 were considered as autism.

The ABC consists in 57 items about the atypical behaviors, and these behaviors are related to five areas (sensory stimuli sensorial; relating; body and object use; language social; self-help). Most items scored from 1 to 4 according to the impairment degree. Children with scores above 53 were considered autism in this study.

### Telomere length measurement

Peripheral blood samples (5 ml) were collected in EDTA tubes and kept at −20°C before use. Genomic DNA was isolated from 200 µl of each blood sample using a commercial DNA Isolation Kit NEP004-1 (Beijing Dingguo Changsheng Biotechnology Co., Ltd., China). The procedure for DNA extraction and purification was performed by using the silica-membrane-based spin column method. The quantity and purity of the DNA was assayed using a Nanodrop 2000 spectrophotometer (Thermo Scientific, Wilmington, DE, USA) and then all the DNA samples were stored at −70°C until use. The RLT was measured by a modified version of the quantitative real-time PCR method originally described by Cawthon^53^. In brief, the real-time PCR was assayed by using Maxima SYBR Green qPCR Master Mix (Thermo Scientific) performed with ABI 7500 Fast Real-Time PCR System (Applied Biosystems, Foster City, CA). The primers for the telomere PCR were 270 nM Tel1 (5′-GGTTTTTGAGGGTGAGGGTGAGGGTGAGGGTGAGGGT-3′) and 900 nM Tel2 (5′-TCCCGACTATCCCTATCCCTATCCCTATCCCTATCCCTA-3′) and for single-copy gene (36B4) PCR were 300 nM 36B4u (5′-CAGCAAGTGGGAAGGTGTAATCC-3′) and 500 nM 36B4d (5′-CCCATTCTATCATCAACGGGTACAA-3′)^53^. The profile of the telomere amplification was 95°C for 10 min followed by 30 cycles of 95°C for 15 sec, 54°C for 2 min and 72°C for 30 sec. The profile of the 36B4 amplification was 95°C for 10 min followed by 35 cycles of 95°C for 15 sec and 60°C for 1 min 10 sec. Each sample was run in triplicate using 10 ng DNA per 10 µl reaction. The telomere and 36B4 PCR reactions were carried out on separate 96-well plates with the same samples in the same well positions. A no-template control and the same calibrator sample were included in each run. Melting curve analysis was performed for every run to verify specificity and a standard curve was produced by a serially diluting reference DNA samples (ranging from 2.5 ng to 20 ng) in every plate to assess the PCR efficiency. The mean slope of the standard curve was -3.24 for both the telomere and 36B4 reactions, and the linear correlation coefficient value for both reactions were 0.98 and 0.99, respectively. The RLT for each sample was examined by determining the ratio (T/S) of telomere repeat copy number (T) relative to 36B4 copy number (S), relative to the same reference sample. The results were expressed by the relative T/S ratios, which were calculated as previously described^53^,^54^,^55^,^56^. The average coefficients of variation (CV) within triplicates were 1.74% for telomere assay and 0.55% for 36B4 assay separately, and the average inter-assay CV for the T/S ratio was 4.75%.

### Statistical analysis

All analyses were performed using the Statistical Package for Social Sciences (SPSS, version 17.0 for Windows). To compare RTL and characteristics between study groups, chi-square test was used for categorical variables (gender) and the Student's t test for continuous variables (age, RTL) when these data were approximately normally distributed. RTL were also analyzed as categorical variables categorized according to the cutoff point at the median and tertile value of the cohort-specific distribution in control group. The association between the RLT and the risk of autism was examined by utilizing unconditional logistic regression, adjusting for age and gender, to calculate the adjusted odds ratio (OR) and 95% confidence interval (CI) and tests for trend was calculated by using the level of RLT as a continuous variable, and the goodness of fit for logistic regression models were assessed by Hosmer–Lemeshow test. Then, Pearson correlation analysis was used to assess the independent effects of age and clinical characteristics on the RLT. All P-values were two-sided and considered statistically significant at <0.05.

## Author Contributions

Z.L., J.T., K.X. and X.C. designed the study. S.C., Z.W. and G.W. collected the samples and participants' characteristics. Z.L., H.L. and Y.H. carried out the experiment. Z.L., J.T., X.X. and Y.L. analyzed and discussed the experimental result. Z.L., J.T. and X.C. wrote the paper. All authors read and approved the final manuscript.

## Figures and Tables

**Table 1:** Characteristics of Cases and Controls

Variable	Cases, (n = 110)	Controls, (n = 129)	P values
Age, months (mean ± SD)	56.98 ± 24.94	59.82 ± 26.06	0.393
Gender (%)			0.008
Male	98 (89.1)	98 (76.0)	
Female	12 (10.9)	31 (24.0)	
Family training (no/yes)*	49/43		
Medication exposure(no/yes)*	79/12		
CARS Score, mean ± SD	34.61 ± 3.09		
ABC Score, mean ± SD	104.35 ± 9.50		
Paternal age, years (mean ± SD)	30.39 ± 4.65		
Maternal age, years (mean ± SD)	27.19 ± 4.38		
Age of onset, years (mean ± SD)	2.16 ± 0.71		
Illness of duration, years (mean ± SD)	3.11 ± 2.11		

*fraction of data missing.

**Table 2:** Associations between telomere length and incident of autism

Telomere length	Cases, n (%)	Controls, n (%)	Adjusted OR (95% CL)*	P value
By median				
Long	36 (32.7)	65 (50.4)	1.00 (reference)	-
Short	74 (67.3)	64 (49.6)	2.15 (1.25–3.71)	0.006
By tertile				
First	21 (19.1)	43 (33.3)	1.00 (reference)	-
Second	35 (31.8)	43 (33.3)	1.66 (0.82–3.36)	0.16
Third	54 (49.1)	43 (33.3)	2.59 (1.31–5.14)	0.006
P for trend**			1.61(1.15–2.25)	0.006

Abbreviations: OR, odds ratio; CI, confidence interval.

*Adjusted by age and gender. ** P value was calculated using relative telomere length as continuous variable.

**Table 3:** Correlations between telomere length and clinical variables in childhood autism

Variables	r	P value
Paternal age	0.01	0.96
Maternal age	0.02	0.88
Age of onset	−0.02	0.88
Illness of duration	−0.08	0.43
CARS Score	−0.01	0.90
ABC Score	−0.04	0.69
